# Detection of clinically important non tuberculous mycobacteria (NTM) from pulmonary samples through one-step multiplex PCR assay

**DOI:** 10.1186/s12866-020-01952-y

**Published:** 2020-08-26

**Authors:** Kamal Singh, Richa Kumari, Rajneesh Tripathi, Smita Gupta, Shampa Anupurba

**Affiliations:** Department of Microbiology, Institute of Medical Sciences, Banaras Hindu University, Varanasi, Uttar Pradesh India

**Keywords:** NTM, Multiplex PCR, MTBC, MOTT, *Mycobacterium avium complex*, *Mycobacterium abscessus* and *Mycobacterium kansasii*

## Abstract

**Background:**

The burden of non-tuberculous mycobacterial (NTM) disease is increasing worldwide but still its diagnosis is delayed and it is mistaken as multidrug-resistant tuberculosis (MDR-TB).The present study was performed to develop a multiplex PCR assay for detection and identification of clinically most common NTM to the species level from pulmonary samples.

**Results:**

Out of 50 isolates, 26 were identified as *Mycobacterium kansasii* (MK), 20 were identified as *Mycobacterium abscessus* (MA) and 4 were identified as *Mycobacterium avium complex* (MAC) through multiplex PCR and further confirmed by sequencing.

**Conclusion:**

Our study showed that multiplex PCR assay is a simple, convenient, and reliable technique for detection and differential identification of major NTM species.

## Background

Tuberculosis (TB), caused by *Mycobacterium tuberculosis* complex (MTBC), persists as the principal killer disease worldwide, notably in the developing countries and has been a major public health problem in spite of considerable progress in diagnosis and treatment [1]. The genus Mycobacterium comprises several species that are divided into three groups, the MTBC, *Mycobacterium leprae* and atypical or non-tuberculous mycobacteria (NTM) [2]. NTM, also known as environmental mycobacteria or mycobacteria other than tuberculosis (MOTT), are mycobacteria which are generally free-living organisms and found ubiquitously in the environment [3]. There has been approximately 200 NTM species identified to-date [4]. They can cause a wide range of infections, with pulmonary infections being the most frequent (65–90%) [5].

Nowadays NTM have become important human pathogens as the incidence and prevalence of disease caused by them continue to increase worldwide [6]. The disease causing agents among NTM differ geographically, but the most common species that are frequently isolated from patients with NTM infection are *Mycobacterium avium complex* (MAC) (*Mycobacterium avium, Mycobacterium intracellulare* and *Mycobacterium chimaera), Mycobacterium abscessus complex* (*Mycobacterium abscessus* subspecies *bolletii*, subspecies *massiliense* and *Mycobacterium chelonae*) and *Mycobacterium kansasii* [7, 8]. The identification and differentiation of NTM from MTBC is of important diagnostic value as the pathogenesis and treatment regimens for these diseases are different [6, 9, 10].The varying pattern of susceptibility towards anti-TB drugs imposes need of different treatment strategies even among the NTM of same species complex [11–13]. Thus, rapid differentiation of MTBC from NTM and species-specific identification of NTM is crucial for proper treatment and appropriate patient management.

Usually, the prevalence of NTM infections has been notified from TB non-endemic countries and rarely from TB endemic countries because the chances of missing NTM infections are higher in TB endemic countries [14, 15]. The current standard of care for diagnostic tests does not include bacterial characterization leading to smear positive NTM cases being misclassified as MTBC. So, majority of NTM infections remain either undetected or receive chemotherapy commonly used for tuberculosis causing evolution of drug resistant NTM strains. Besides cases of mixed infection have also been reported by a few workers [16, 17].Mostly these NTM species are identified by phenotypic methods which are very cumbersome and time taking. Thus the study was designed to develop a multiplex PCR assay for detection and identification of clinically most common NTM to the species level from pulmonary samples.

## Result

**Fig. 1:**
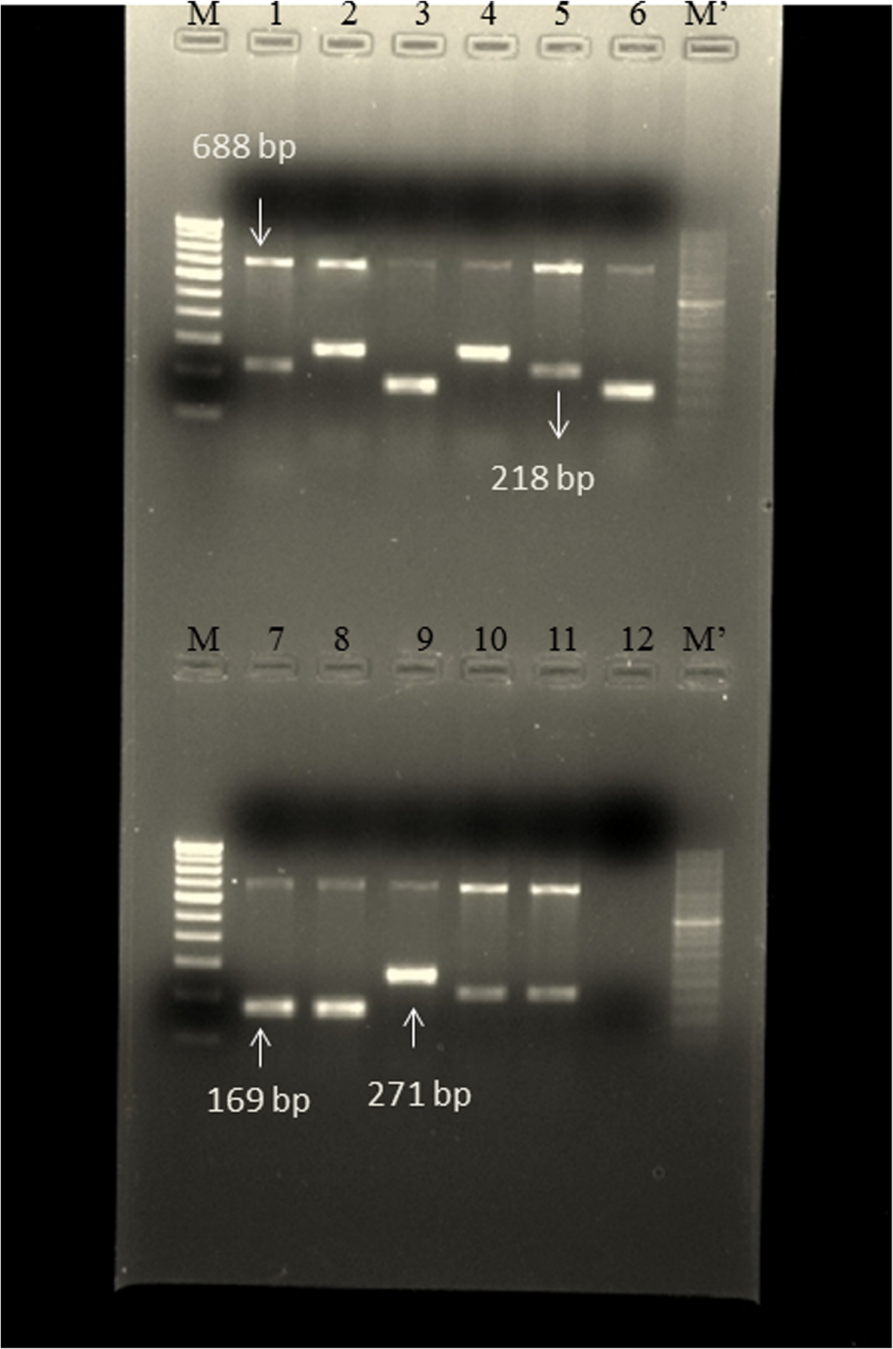
Amplified PCR product. Image showing amplified products of *16S rRNA* and *ITS* region gene M: Marker 100 bp; M’: Marker 50 bp; Lane1,2 & 3; controls of MK*,* MA *& MAC*. Lane 5, 10 & 11 positive band for MK; Lane 4 & 9: positive bands for MA; Lane 6,7 & 8: positive bands for MAC; Lane 12: negative control

**Fig. 2:**
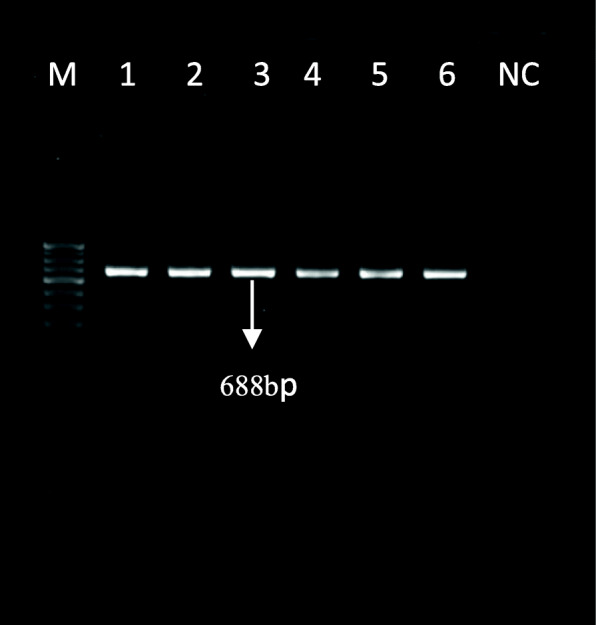
Amplified PCR product. Image showing genus specific (*16S rRNA)* bands M: Marker 100 bp; Lane1; *Mycobacterium tuberculosis* (H37Rv), Lane 2; *Mycobacterium fortuitum*; Lane 3, 4, 5 & 6: clinically confirmed MTBC; NC: negative control

In this study, there were a total of 50 cultures which were smear positive but negative by capilia and GenoType MTBDR *plus* Assay. The multiplex PCR using four sets of primers for three unrelated mycobacterial species was successfully developed. At first multiplex PCR was tested with control strains of *Mycobacterium avium complex* (MAC), *Mycobacterium kansasii* (MK) and *Mycobacterium abscessus* (MA)*.* In each strain of MAC, MK and MA we found two bands, one genus specific 688 bp and another band of 169 bp, 218 bp and 271 bp respectively for each species as shown in Fig. 1.In *Mycobacterium tuberculosis*(H37Rv), clinically confirmed MTBC and known strain of *Mycobacterium fortuitum* only one genus specific (688 bp) band was found as shown in Fig. 2. Out of 50 isolates, 26 were identified as *Mycobacterium kansasii*, 20 were identified as *Mycobacterium abscessus* and 4 were identified as *Mycobacterium avium complex* through multiplex PCR and further confirmed by sequencing. The sequencing data of control strains and other isolates were analyzed with the help of Basic Local Alignment Search Tool (BLAST) which finds regions of local similarity between the Internal transcribed spacer (ITS) region sequences of reference strains such as *Mycobacterium avium complex:* accession no*.*CP040255.1*, Mycobacterium abscessus:* accession no*.*CP030860.1*, Mycobacterium kansasii:* accession no*.*LR031424.1.

## Discussion

In the current study, we identified the most common NTM species from pulmonary samples. Out of these NTM species 26 (52%) were identified as *Mycobacterium kansasii*, 20 (40%) *Mycobacterium abscessus* and 4(8%) as *Mycobacterium avium complex* by multiplex PCR and further confirmed by sequencing.

Based on only phenotypic characterization or combined with hybridization with DNA probes, the commonest species isolated from pulmonary specimens in earlier studies has been MAC. The other species were *Mycobacterium abscessus, Mycobacterium xenopi*, *Mycobacterium kanasii, Mycobacterium chelonae* and *Mycobacterium fortuitum* although their prevalence varied [18–23]. In contrast Wang HX, et al.*,* [24] reported *Mycobacterium chelonae* (26.7%), followed by *Mycobacterium fortuitum* (15.4%), *Mycobacterium kansasii* (14.2%), *Mycobacterium avium-intracellulare complex* (13.1%) and *Mycobacterium terrae* (6.9%) which were identified with conventional biochemical tests and 16S rRNA gene sequencing in suspected pulmonary and extra pulmonary tuberculosis [24].

A study from Singapore reported the identification and differentiation of clinically relevant NTM species with two sets of multiplex PCR targeting the ITS region. They found *Mycobacterium abscessus* (26.3%) followed by *Mycobacterium fortuitum* (24.5%) and *Mycobacterium avium–intracellulare complex* (18.2%) [25]. According to Ito Y, et al.*,* [26], MAC was most frequently isolated (85.9%), followed by *Mycobacterium abscessus* (2.8%) and *Mycobacterium kansasii* (1.2%) with the help of molecular techniques like COBAS Amplicor PCR assay, COBAS TaqMan MAI test (Roche Diagnostics, Basel, Switzerland) and DNA-DNA hybridization with the DDH Mycobacteria Kit (Kyokuto Pharmaceutical Industrial Co., Tokyo, Japan) [26]. A study from Saudi Arabia showed that the incidences of NTM causing pulmonary and extrapulmonary diseases were reportedly increasing and most prominent species were *Mycobacterium simiae* (22.6%), *Mycobacterium fortuitum* (18.1%), *Mycobacterium abscessus* (17.8%), MAC (11.2%) and *Mycobacterium kansasii* (3.7%). Primary species identification was carried out by line probe assays followed by sequencing [27]. Hu C, et al.*,* [28] observed five different species of NTM causing pulmonary disease with the help of Mycobacterium Species Identification kit (PCR-reverse dot blot) of DaAn Gene company, such as *Mycobacterium intracellulare* (70.1%), *Mycobacterium abscessus* (11.5%) and *Mycobacterium avium* (11.5%) of isolates. A small number of cases were due to *Mycobacterium kansasii*, (7.5%) and *Mycobacterium gordonae* (1.1%) [28].

In a study from India NTM often associated with pulmonary and extrapulmonary disease, which are identified by PCR restriction analysis (PRA) of the *hsp65* gene, that included *Mycobacterium chelonae* (28.97%), *Mycobacterium fortuitum* (19.62%), *Mycobacterium avium* complex (17.75%), *Mycobacterium gordonae* (11.21%), *Mycobacterium terrae* complex (8.41%), *Mycobacterium kansasii* (3.73%), *Mycobacterium scrofulaceum* (2.80%), *Mycobacterium simiae* (2.80%), *Mycobacterium ulcerans* (1.86%), *Mycobacterium abscessus* (0.93%), *Mycobacterium malmoense* (0.93%) and *Mycobacterium phlei* (0.93%) [29]. Jain S, et al.*,* [30] found that the most common NTM species from pulmonary and extrapulmonary samples were *Mycobacterium kansasii* (30.1%) *Mycobacterium chelonae* (23.3%), *Mycobacterium xenopi* (15.4%), *Mycobacterium scrofulaceum* (7.8%)*, Mycobacterium avium* (7.8%)*, Mycobacterium asiaticum* (7.8%)*,* and *Mycobacterium fortuitum* (7.8%) based on biochemical tests. Further they performed multiplex PCR using different primers for Mycobacterium genus (targeting hsp65), *Mycobacterium tuberculosis complex* (targetingESAT6), and *Mycobacterium avium complex* (targeting MAC) specific genes. So MAC was the only NTM which was identified by multiplex PCR [30]. A study by Sharma P, et al.*,* [31] reported that NTM isolated from pulmonary samples were *Mycobacterium intracellulare* (62.5%), *Mycobacterium flavescens* (12.5%), *Mycobacterium genavense* (12.5%), and *Mycobacterium gordonae* (12.5%) whereas extrapulmonary NTM isolates included *Mycobacterium intracellulare* (6.5%)*, Mycobacterium abscessus* (2.6%)*, Mycobacterium avium* (1.3%)*, Mycobacterium mucogenicum* (1.3%)*, Mycobacterium austroafricanum* (1.3%), and *Mycobacterium gordonae* (10.4%). These were identified by PRA and gene sequencing [31].

Thus it can be seen that spectrum of NTM varied in different geographical regions. Molecular tools like multiplex PCR or PRA can help in rapid identification. However, PRA is costlier compared to multiplex PCR. The limitation of this study is that it includes only fifty isolates. Multiplex PCR has disadvantages that it couldn’t include more number of primers because primers inhibit each other. However, all fifty isolates could be identified by this multiplex PCR.

## Conclusion

In conclusion, multiplex PCR is a simple, fast, convenient and reliable technique for identification of NTM species in the routine laboratory. This method can be used in developing countries for identification of most common NTM from pulmonary samples. To the best of our knowledge this is the first type of study conducted in India.

## Methods

### Study design and identification of isolates

This study was undertaken in the Department of Microbiology, Institute of Medical Sciences, Banaras Hindu University, at Varanasi. It is the extension of our previous work [32] where out of 60 positive cultures, 10 (16.7%) were found positive by both GenoType MTBDR plus assay (LPA) and PCR but remaining 50 which were liquid culture (MGIT 960) positive, but capilia and LPA negative were included in this study. Control strains of *Mycobacterium avium complex* (MAC), *Mycobacterium kansasii* (MK) and *Mycobacterium abscessus* (MA) were obtained from National Reference Laboratory, National Institute of Tuberculosis and Respiratory Diseases, New Delhi, India.

### DNA extraction

DNA isolation from the positive MGIT cultures as well as solid culture was done by CTAB-chloroform method with some modifications in BSL-3 laboratory [33, 34]. The quality and quantity of DNA were analyzed by a spectrophotometer (Thermo Scientific NanoDrop 2000).

### Primer designing for the study

At first sequence of *Mycobacterium tuberculosis, Mycobacterium avium complex, Mycobacterium kansasii* and *Mycobacterium abscessus* was downloaded from National Center for Biotechnology Information (NCBI) data base. Then the genus specific primer was designed from 16S rRNA region which is specific for *Mycobacterium* genus. The species specific primer from the Internal transcribed spacer (ITS) region of *Mycobacterium avium complex, Mycobacterium abscessus* and *Mycobacterium kansasii* were designed. Annealing temperature and GC content were calculated for both forward and reverse complementary primer using Tm calculator Thermo Fisher Scientific software. The detail of prepared primers is enlisted in Table 1.

**Table 1:** Oligonucleotide used as primer for amplification

S. No.	Target Gene	Target Organism	Primer Sequences	Product Size (bp)	Reference
**1**	16S rRNA	Mycobacterium Species	TGAGATACGGCCCAGACTCCT CTCTAGACGCGTCCTGTGCAT	688	This study
**2**	ITS region	MAC	CAACAGCAAATGATTGCCAG CACATTTCGATGAACGCCG	169	This study
**3**	ITS region	MK	ATCCCAACAAGTGGGGTGC CGCTACCCGTAGGGCAACG	218	This study
**4**	16S rRNA	MA	CCTTTCTAAGGAGCACCATTT CGAGCGAGGCTATGTTTAGAT	271	This study

### Development of multiplex PCR for identification of different NTM species

Initially, all fifty isolates were screened individually by targeting ITS region (MAC, MA and MK) sequences which were species-specific. This helped us to identify all the isolates at species level. Further gradient multiplex PCR was performed with 16S rRNA genus-specific primer and ITS (MAC, MA and MK) species-specific primers to know the primer working conditions. On the basis of results obtained in gradient multiplex PCR, the in house multiplex PCR was developed. This multiplex PCR targeted 16S rRNA (genus specific) and ITS (MAC, MA and MK) sequences as shown in Table 1. Different reaction mixtures were added in the PCR tube and run in thermal cycler at the amplifying conditions as shown in Table 2. The reference strain MAC, MK and MA were used as positive controls and PCR grade water was used as a negative control. The multiplex PCR was also tested against standard strain of *Mycobacterium tuberculosis* (H37Rv), clinically confirmed MTBC and known strain of *Mycobacterium fortuitum.*

**Table 2:** PCR master mix 25 μl volume

Sr. No.	Constituent	Con.^**n**^	For 1 reaction (μl)
1	*Taq* buffer	10X (GeNei)	2.5
2	dNTP Mix	200 M (GeNei)	2.0
3	*Taq*Polymerase	5 unit	0.3
4	*16S rRNA* forward	10 μM	1
5	*16S rRNA* reverse	10 μM	1
6	MAC forward	10 μM	1
7	MAC reverse	10 μM	1
8	MK forward	10 μM	1
9	MK reverse	10 μM	1
10	MA forward	10 μM	1
11	MA reverse	10 μM	1
12	DNA		5
13	Deionised water		7.2

### Multiplex PCR running conditions

Initial denaturation step at 95 °C for 15 min followed by following parameters:

$$ \left.\begin{array}{l}\mathrm{DNA}\ \mathrm{denaturation}\ \mathrm{at}\ {95}^{\mathrm{o}}\mathrm{C}\ \mathrm{for}\ 1\ \mathrm{minute}\\ {}\mathrm{Primer}\ \mathrm{annealing}\ \mathrm{at}\ 61.{5}^{\mathrm{o}}\mathrm{C}\ \mathrm{for}\ 1\ \mathrm{minute}\\ {}\mathrm{Extension}\ \mathrm{at}\ {72}^{\mathrm{o}}\mathrm{C}\ \mathrm{for}\ 1\ \mathrm{minute}\end{array}\right\}30\ \mathrm{cycles} $$

Final extension step at 72 °C for 10 min.

### Sequencing

The ITS region was amplified with the help of primers for MAC, MK and MA. Product size was confirmed by agarose (2%) gel electrophoresis. Four representative strains from each species were sent for sequencing.

### Sequence data analysis

The sequencing data of control strains and other isolates were analyzed with the help of Basic Local Alignment Search Tool (BLAST). BLAST finds regions of local similarity between sequences [35].

## Data Availability

The datasets generated and/or analyzed during the current study are not publicly available due confidentiality agreement at the department of microbiology, Institute of Medical Sciences, Banaras Hindu University but are available from the corresponding author on reasonable request.

## Abbreviations

NTM: Non-tuberculous mycobacteria
MGIT: Mycobacterium Growth Indicator Tube
TB: Tuberculosis
MTBC: *Mycobacterium tuberculosis* complex
MOTT: Mycobacterium other than tuberculosis
MAC: *Mycobacterium avium complex*
MK: *Mycobacterium kansasii*
MA: *Mycobacterium abscessus*
LPA: Line Probe Assay
NCBI: National center for biotechnology information
ITS: Internal transcribed spacer
BLAST: Basic local alignment search tool
PRA: PCR restriction analysis
